# Publication trends of Allergy, Pediatric Allergy and Immunology, and Clinical and Translational Allergy journals: a MeSH term-based bibliometric analysis

**DOI:** 10.1186/s13601-018-0191-1

**Published:** 2018-02-22

**Authors:** Daniel Martinho-Dias, Bernardo Sousa-Pinto, Júlio Botelho-Souza, António Soares, Luís Delgado, João Almeida Fonseca

**Affiliations:** 1 MEDCIDS, Department of Community Medicine, Information and Health Decision Sciences, Faculty of Medicine, University of Porto, Rua Dr. Placido da Costa, 4200-450 Porto, Portugal; 2 CINTESIS, Center for Health Technology and Services Research, Rua Dr. Placido da Costa, 4200-450 Porto, Portugal; 3 Basic and Clinical Immunology Unit, Department of Pathology, Faculty of Medicine, University of Porto, Porto, Portugal

**Keywords:** Bibliometrics, Medical subject headings, Research trends

## Abstract

We performed a MeSH term-based bibliometric analysis aiming to assess the publication trends of EAACI journals, namely Allergy, Pediatric Allergy and Immunology (PAI) (from 1990 to 2015) and Clinical and Translational Allergy (CTA) (from its inception in 2011 to 2015). We also aimed to discuss the impact of the creation of CTA in the publication topics of Allergy and PAI. We analysed a total of 1973 articles and 23,660 MeSH terms. Most MeSH terms in the three journals fell in the category of “basic immunology and molecular biology” (BIMB). During the studied period, we observed an increase in the proportion of MeSH terms on BIMB, and a decreasing proportion of terms on allergic rhinitis and aeroallergens. The observed changes in Allergy and PAI publication topics hint at a possible impact from CTA creation.

**Electronic supplementary material:**

The online version of this article (10.1186/s13601-018-0191-1) contains supplementary material, which is available to authorized users.

To the editor

Bibliometric methods are used to quantitatively analyse published scientific literature for potential identification of relevant patterns [1, 2]. We performed a MeSH term-based bibliometric analysis of publication topics of the three EAACI journals, namely Allergy, Pediatric Allergy and Immunology (PAI), and Clinical and Translational Allergy (CTA). We aimed to assess the evolution of publication trends in the fields of Allergy/Immunology, and to discuss whether the creation of CTA (in 2011) associated with changes in the publication topics by Allergy and PAI.

We assessed original and review articles published in Allergy, PAI, and CTA. For each year between 1990 (first year of PAI) and 2015, we randomly selected and analysed 20% of Allergy publications and 40% of PAI publications. Additionally, we assessed all CTA publications until 2015. We retrieved the title, abstract and keywords of selected publications—keywords were converted into MeSH terms via Unified Medical Language System^®^ terminology, while titles and abstracts were text-mined to obtain further MeSH terms. This was achieved using the online tool Syn4Data (http://www.syn4data.med.up.pt/). Two independent researchers verified the obtained results and excluded inadequate/wrongly attributed or generalist/uninformative MeSH terms (Additional file 1: Table 1).

For each journal, we obtained the frequency of publications to which each MeSH term was attributed. MeSH terms were categorised into nine “thematic” categories—(1) asthma and lower airways diseases, (2) allergic rhinitis and allergy to aeroallergens, (3) food allergy and nutrition, (4) drug allergy, (5) anaphylaxis and insect venom allergy, (6) skin and eye diseases, (7) diagnosis methods, (8) asthma and allergy therapy, and (9) basic immunology and molecular biology (BIMB). For each journal, we assessed the time trends of each category, performing a univariable logistic regression with time (in years) as independent variable.

We then compared the proportions of each category terms between the three journals. To address whether CTA had any influence in the topics published by Allergy and PAI, we compared MeSH terms proportions in these latter two journals prior and after 2011. Multivariable logistic regressions were performed for each category, adjusting for changes of the Editors-in-chief. Similar comparisons were performed concerning the proportions of publications assessing animal models.

We assessed 23,660 MeSH terms from 1973 articles. Most publications (*n* = 1001) and MeSH terms (*n* = 11,788) were from Allergy, followed by PAI (791 publications; 10,260 terms) and CTA (181 publications; 1612 terms). Allergy and CTA had significantly more publications with animal models (12.5 and 11.6%, respectively) than PAI (5.6%) (*p* < 0.001 and *p* = 0.003, respectively). More post-2011 than pre-2011 Allergy publications assessed animal models (19.3 vs. 10.8%; *p* = 0.001).

Within the three journals altogether, significant increases over time were observed for proportion of MeSH terms on “food allergy and nutrition” [OR 1.03 per year (95% CI 1.02–1.04); *p* < 0.001], “skin and eye diseases” [OR 1.04 per year (95% CI 1.03–1.05); *p* < 0.001], and “BIMB” [OR 1.01 per year (95% CI 1.00–1.01); *p* = 0.003] (Fig. 1a; Additional file 1: Table 2A). Most MeSH terms in all journals concerned BIMB, comprising 41.6% of Allergy terms, versus 33.5% of PAI (*p* < 0.001) and 29.5% of CTA (*p* < 0.001). In Allergy, more terms belonged to BIMB category after than before 2011 (51.0 vs. 38.8%; *p* < 0.001), even after adjustment for Editorial changes [OR 1.37 (95% CI 1.09–1.73); *p* = 0.006]. In PAI, the opposite trend was observed (35.8 post-2011 vs. 26.9% pre-2011; *p* < 0.001), even after adjusting for the Editors-in-chief [OR 0.62 (95% CI 0.51–0.75); *p* < 0.001] (Fig. 1b; Additional file 1: Table 2B).

**Fig. 1:**
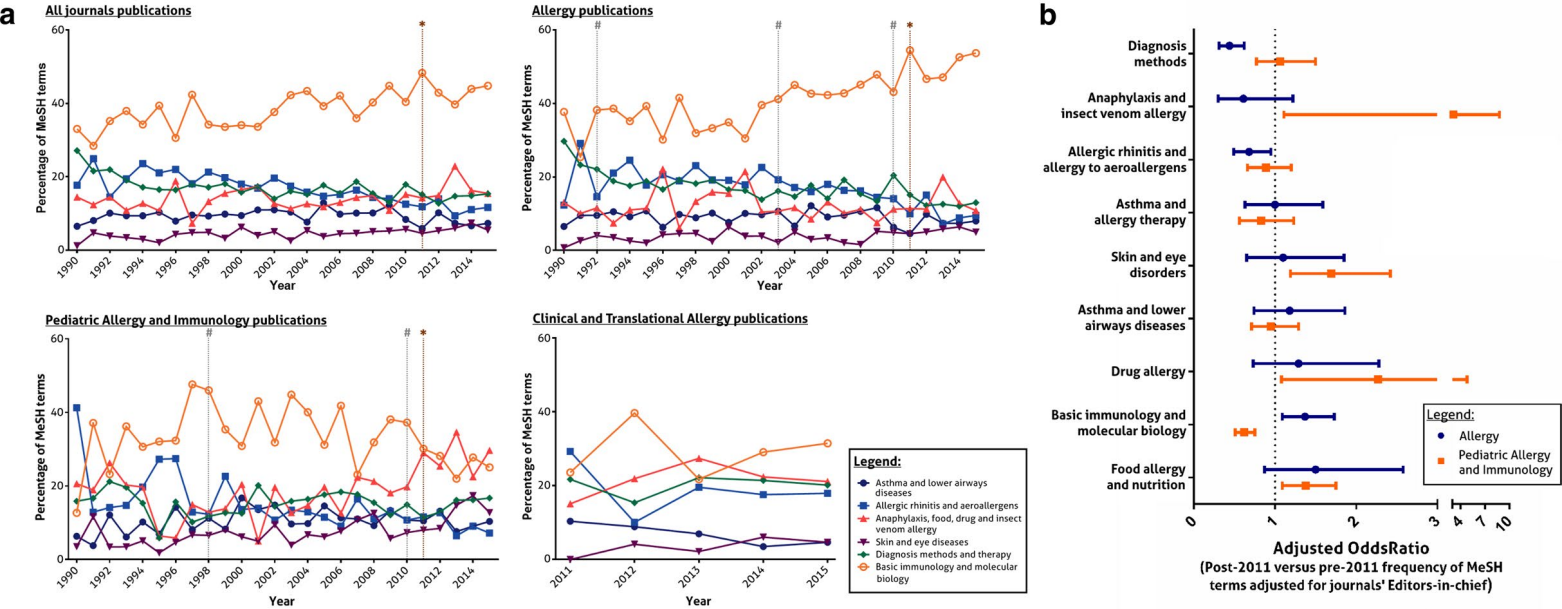
**a** Annual percentages of MeSH terms of each thematic category (excluding “others”) among Allergy, Pediatric Allergy and Immunology, and Clinical and Translational Allergy (CTA) journals [the creation of CTA is marked with a star (*), while changes in the Editor-in-chief are marked with hashes (#)]. **b** Comparison of pre- and post-2011 MeSH terms (2011 corresponds to the year of creation of CTA) of each thematic category (excluding “others”) among Allergy and Pediatric Allergy and Immunology journals, after adjustment for the journals Editors-in-chief

On the contrary, post-2011 Allergy publications had lower proportion of terms on “diagnosis methods” (6.7 vs. 10.7%; *p* < 0.001), “asthma and lower airways diseases” (7.4 vs. 9.4%; *p* = 0.003), and “allergic rhinitis and allergy to aeroallergens” (18.6 vs. 10.1%; *p* < 0.001); this latter decrease was significant even after adjusting for Editorial changes [OR 0.68 (95% CI 0.49–0.95); *p* = 0.024]. The proportion of “allergic rhinitis and allergy to aeroallergens” terms was also lower in post- than pre-2011 PAI articles (14.4 vs. 9.7%; *p* < 0.001). Consistently, within the three journals altogether, there were significant decreases over time in the percentage of MeSH terms on “diagnosis methods” [OR 0.98 per year (95% CI 0.98–0.99); *p* < 0.001] and “allergic rhinitis and allergy to aeroallergens” [OR 0.97 per year (95% CI 0.96–0.97); *p* < 0.001].

In conclusion, this is the first bibliometric study assessing publication trends in the Allergy/Immunology field, although restricted to EAACI journals. Its keyword-based approach maximised the information extracted from publications’ metadata [3]. The observed changes in Allergy and PAI post-2011 publication topics suggest a possible influence from CTA creation. Nevertheless, we did not assess other potentially relevant aspects, namely the existence and effect of other non-EAACI competing journals with a similar scope, the journals’ impact factors, and the scientific and clinical background of the Editors-in-chief (we only adjusted for Editorial changes). Overall, during the studied period, we observed an increase in the proportion of MeSH terms on basic immunology, “skin and eye diseases” and “food allergy and nutrition”, and a decreasing proportion of terms concerning “allergic rhinitis and aeroallergens” and “diagnosis methods”. This analysis provides a starting point for discussing publication and research trends in the Allergy/Immunology field. Future studies should assess a larger set of journals in the field.

## Additional file

**Additional file 1: Table 1.** List of excluded generalist MeSH terms in the context of an analysis in the field of allergy and immunology. **Table 2**. Temporal trends of MeSH terms of each thematic category (results obtained by univariable logistic regression, with the year of publication as independent variable) (A), and absolute and relative frequencies of MeSH terms of each thematic category (B) among Allergy, Pediatric Allergy and Immunology (PAI), and Clinical and Translational Allergy (CTA) journals.

## Abbreviations

BIMB: basic immunology and molecular biology
CTA: Clinical and Translational Allergy
EAACI: European Academy of Allergy and Clinical Immunology
OR: odds ratio
PAI: Pediatric Allergy and Immunology
