# Supplementary material for: Publication trends of Allergy, Pediatric Allergy and Immunology, and Clinical and Translational Allergy journals: a MeSH term-based bibliometric analysis
Source: Clin Transl Allergy. 2018 Feb 22;8:6. doi: 10.1186/s13601-018-0191-1 (PMC5822487; doi:10.1186/s13601-018-0191-1)
Supplement: Supplementary file 1 — Additional file 1: Table 1. List of excluded generalist MeSH terms in the context of an analysis in the field of allergy and immunology. Table 2. Temporal trends of MeSH terms of each thematic category (results obtained by univariable logistic regression, with the year of publication as independent variable) (A), and absolute and relative frequencies of MeSH terms of each thematic category (B) among Allergy, Pediatric Allergy and Immunology (PAI), and Clinical and Translational Allergy (CTA) journals. [file 13601_2018_191_MOESM1_ESM.docx]

**Additional tables**

**Additional Table 1.**

| MeSH terms considered generalist |
| --- |
| - Allergens - Allergy and Immunology - Biological Factors - Disease - Health - Hypersensitivity - Immune System - Immune System Diseases - Immune System Processes - Immunity - Immunologic Factors |

Legend: List of excluded generalist MeSH terms in the context of an analysis in the field of allergy and immunology.

**Additional Table 2.**

| A. Temporal trends of MeSH terms of each thematic category^a^ | | | | | | | | | | | | |
| --- | --- | --- | --- | --- | --- | --- | --- | --- | --- | --- | --- | --- |
|  | **Journals – OR *per* year (95%CI); *p* value** | | | | | | | | | | | |
|  | **Allergy** | | | |  | **PAI** | | | |  | **CTA** | **All journals** |
| Asthma and lower airways diseases | 0.99 (0.98-1.00); 0.148 | | | |  | 1.00 (0.99-1.01); 0.599 | | | |  | 0.77 (0.65-0.92); 0.003 | 0.99 (0.99-1.00); 0.084 |
| Allergic rhinitis and allergy to aeroallergens | 0.96 (0.96-0.97); <0.001 | | | |  | 0.96 (0.95-0.97); <0.001 | | | |  | 0.96 (0.86-1.07); 0.473 | 0.97 (0.96-0.97); <0.001 |
| Food allergy and nutrition | 0.99 (0.98-1.00); 0.048 | | | |  | 1.04 (1.03-1.05); <0.001 | | | |  | 1.00 (0.89-1.13); 0.964 | 1.03 (1.02-1.04); <0.001 |
| Drug allergy | 1.02 (1.00-1.03); 0.039 | | | |  | 1.03 (1.00-1.06); 0.062 | | | |  | 1.13 (0.87-1.48); 0.360 | 1.01 (1.00-1.02); 0.092 |
| Anaphylaxis and insect venom allergy | 0.99 (0.97-1.02); 0.609 | | | |  | 1.05 (1.01-1.09); 0.010 | | | |  | 0.99 (0.76-1.30); 0.947 | 1.02 (1.00-1.04); 0.074 |
| Skin and eye diseases | 1.02 (1.01-1.04); 0.002 | | | |  | 1.05 (1.03-1.07); <0.001 | | | |  | 1.31 (1.02-1.67); 0.032 | 1.04 (1.03-1.05); <0.001 |
| Diagnosis methods | 0.97 (0.96-0.98); <0.001 | | | |  | 1.00 (0.99-1.01); 0.542 | | | |  | 0.94 (0.82-1.08); 0.375 | 0.98 (0.98-0.99); <0.001 |
| Asthma and allergy therapy | 0.99 (0.98-1.00); 0.037 | | | |  | 1.00 (0.99-1.02); 0.819 | | | |  | 1.12 (0.96-1.30); 0.150 | 1.00 (0.99-1.01); 0.979 |
| Basic immunology and molecular biology | 1.04 (1.03-1.04); <0.001 | | | |  | 0.98 (0.98-0.99); <0.001 | | | |  | 1.02 (0.93-1.13); 0.625 | 1.01 (1.00-1.01); 0.003 |
| B. Frequencies of MeSH terms of each thematic category^a^ | | | | | | | | | | | | |
|  | **Allergy – *n* of MeSH terms - % (SE); 95%CI** | | | |  | **PAI – *n* of MeSH terms - % (SE); 95%CI** | | | |  | **CTA (2011-2015) – *n* of MeSH terms - % (SE); 95%CI** | **All journals – *n* of MeSH terms** |
|  | **Total** | **1990-2010** | **2011-2015** | ***p* value^b^** |  | **Total** | **1990-2010** | **2011-2015** | ***p* value^b^** |  |  |  |
| Asthma and lower airways diseases | 866 - 8.9 (0.3); 8.4-9.5 | 703 - 9.4 (0.3); 8.7-10.1 | 163 - 7.4 (0.6); 6.3-8.4 | 0.003 |  | 810 – 11.0^c^ (0.4); 10.2-11.7 | 611 - 11.2 (0.4); 10.3-12.0 | 199 - 10.3 (0.7); 8.9-11.6 | 0.282 |  | 72 - 5.9^c,d^ (0.7); 4.6-7.2 | 1744 |
| Allergic rhinitis and allergy to aeroallergens | 1613 - 16.6 (0.4); 15.9-17.4 | 1390 - 18.6 (0.4); 17.7-19.5 | 223 - 10.1 (0.6); 8.8-11.3 | <0.001 |  | 975 - 13.2^c^ (0.4); 12.4-14.0 | 788 - 14.4 (0.5); 13.5-15.4 | 187 - 9.7 (0.7); 8.4-11.0 | <0.001 |  | 221 - 18.0^d^ (1.1); 15.9-20.2 | 2783 |
| Food allergy and nutrition | 680 - 7.0 (0.3); 6.5-7.5 | 540 - 7.2 (0.3); 6.6-7.8 | 140 - 6.3 (0.5); 5.3-7.3 | 0.143 |  | 1246 - 16.8^c^ (0.4); 16.0-17.7 | 801 - 14.7 (0.5); 13.7-15.6 | 445 - 23.0 (1.0); 21.1-24.9 | <0.001 |  | 204 - 16.7^c^ (1.1); 14.6-18.7 | 2119 |
| Drug allergy | 372 - 3.8 (0.2); 3.5-4.2 | 258 - 3.5 (0.2); 3.0-3.9 | 114 - 5.1 (0.5); 4.2-6.1 | <0.001 |  | 136 - 1.8^c^ (0.2); 1.5-2.1 | 78 - 1.4 (0.2); 1.1-1.7 | 58 - 3.0 (0.4); 2.2-3.8 | <0.001 |  | 36 - 2.9^e^ (0.5); 2.0-3.9 | 542 |
| Anaphylaxis and insect venom allergy | 150 - 1.6 (0.1); 1.3-1.8 | 111 - 1.5 (0.1); 1.2-1.8 | 39 - 1.8 (0.3); 1.2-2.3 | 0.357 |  | 77 - 1.0^f^ (0.1); 0.8-1.3 | 37 - 0.7 (0.1); 0.5-0.9 | 40 - 2.1 (0.3); 1.4-2.7 | <0.001 |  | 32 - 2.6^d,g^ (0.5); 1.7-3.5 | 259 |
| Skin and eye diseases | 389 - 4.0 (0.2); 3.6-4.4 | 271 - 3.6 (0.2); 3.2-4.0 | 118 - 5.3 (0.5); 4.4-6.3 | <0.001 |  | 551 - 7.4^c^ (0.3); 6.9-8.0 | 346 - 6.3 (0.3); 5.7-7.0 | 205 - 10.6 (0.7); 9.2-12.0 | <0.001 |  | 50 - 4.1^d^ (0.6); 3.0-5.2 | 988 |
| Diagnosis methods | 946 - 9.8 (0.3); 9.2-10.4 | 797 - 10.7 (0.4); 10.0-11.4 | 149 - 6.7 (0.5); 5.7-7.8 | <0.001 |  | 723 - 9.8 (0.3); 9.1-10.5 | 542 - 9.9 (0.4); 9.1-10.7 | 181 - 9.4 (0.7); 8.1-10.7 | 0.478 |  | 127 - 10.4 (0.9); 8.7-12.1 | 1791 |
| Asthma and allergy therapy | 647 - 6.7 (0.3); 6.2-7.2 | 507 - 6.8 (0.3); 6.2-7.4 | 140 - 6.3 (0.5); 5.3-7.3 | 0.442 |  | 404 - 5.5^c^ (0.3); 4.9-6.0 | 306 - 5.6 (0.3); 5.0-6.2 | 98 - 5.1 (0.5); 4.1-6.0 | 0.377 |  | 122 - 10.0^c,d^ (0.9); 8.3-11.6 | 1168 |
| Basic immunology and molecular biology | 4029 - 41.6 (0.5); 40.6-42.6 | 2899 - 38.8 (0.6); 37.7-39.9 | 1130 - 51.0 (1.1); 48.9-53.1 | <0.001 |  | 2474 - 33.5^c^ (0.5); 32.4-34.5 | 1954 - 35.8 (0.6); 34.5-37.0 | 520 - 26.9 (1.0); 24.9-28.9 | <0.001 |  | 361 - 29.5^c,f^ (1.3); 26.9-32.0 | 6856 |

SE = standard error; CI = confidence interval; ^a^ MeSH terms which could not be classified into any of these categories were excluded from this analysis; ^b^ *p* value for the comparison between 1990-2010 and 2011-2015 proportions; ^c^ *p*<0.001 in comparison with Allergy publications for the same category; ^d^ *p*<0.001 in comparison with PAI publications for the same category; ^e^ *p*=0.011 in comparison with PAI publications for the same category; ^f^ *p*=0.004 in comparison with PAI publications for the same category; ^g^ *p* =0.006 in comparison with Allergy publications for the same category.

Legend: Temporal trends of MeSH terms of each thematic category (results obtained by univariable logistic regression, with the year of publication as independent variable) (A), and absolute and relative frequencies of MeSH terms of each thematic category (B) among Allergy, Pediatric Allergy and Immunology (PAI) and Clinical and Translational Allergy (CTA) journals.
